# A pulmonary aneurysm: don't forget Hughes-Stovin syndrome

**DOI:** 10.11604/pamj.2015.20.445.5938

**Published:** 2015-04-30

**Authors:** Madiha Mahfoudhi, Sami Turki

**Affiliations:** 1 Service de Médecine Interne A, Hôpital Charles Nicolle, Tunis, Tunisie

**Keywords:** Pulmonary aneurysm, hemoptysis, Hughes-Stovin syndrome

## Image in medicine

Hughes-Stovin syndrome is a scarce pathology associating pulmonary artery aneurysms and deep venous thrombosis and affecting commonly the young patient. A 27 year old man was hospitalised for recurrent hemoptysis and a left femoral vein thrombosis. Besides, he had oral ulcers. The ophthalmological examination was normal. Laboratory studies found a microcytic hypochromic anemia (hemoglobin: 11g/dl). The coagulation tests, the renal and the hepatic functions were normal. Besides, the bacteriological examination in search for mycobacterium tuberculosis and the immunological investigations were negative. The chest radiograph revealed a left surrounded para-hilar opacity. The CT of the chest confirmed the presence of a giant aneurysm in the left lower lobe pulmonary artery of 90 mm/72 mm, partially thrombosed, occupying the quasi-totality of the left pulmonary field. All abnormalities of hemostasis, a tumoral origin, a vasculitis like Behçet's disease and an infectious etiology were eliminated in our patient. Therefore, Hughes-Stovin syndrome was our diagnosis. The patient was put on oral corticosteroid (1 mg/kg/day), and intravenous cyclophosphamide; the steroids were subsequently tapered and withdrawn after 6 months until reaching a minimal dose of 10 mg/day. There has been no recurrence of deep venous thrombosis or hemoptysis. There was no evidence of enlargement of the pulmonary artery aneurysms on chest CT scan control. He was programmed for an embolization because of the giant aspect of the aneurysm.

**Figure 1:**
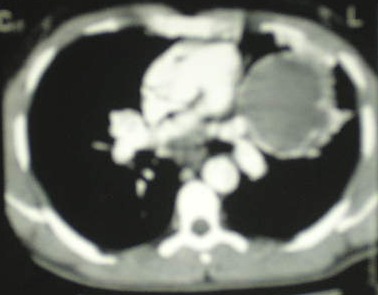
Chest's CT: a giant aneurysm in the left lower lobe pulmonary artery, with mural thrombus

